# Policy communities, devolution and policy transfer: The case of alcohol pricing in Wales

**DOI:** 10.1080/13597566.2021.1934454

**Published:** 2021-06-03

**Authors:** Matthew Lesch, Jim McCambridge

**Affiliations:** Department of Health Sciences, Mental Health and Addiction Research Group, University of York, York, UK

**Keywords:** Policy transfer, devolution, policy learning, multi-level governance, alcohol policy, institutional analysis

## Abstract

This study investigates how processes of horizontal policy transfer can unfold in the context of devolution, examining the development of legislation on minimum unit pricing (MUP) in Wales, following on from Scotland’s earlier policy decision. The study draws on a range of sources, including primary documents, media coverage, and interviews with policy participants. Our analysis identifies the importance of the specific character of Welsh political institutions, particularly the emphasis given to participation and consultation in policymaking. In the case of MUP, we document a process of policy-oriented learning, where policymakers made a concerted effort to draw on an assortment of expertise and experiences, including but not limited to the Scottish model. We also find that the Welsh public health policy community was well placed to support the framing of MUP and to address limitations in policy capacity. The findings hold implications for future studies of learning, devolution, and alcohol policy more generally.

In recent years, evidence-informed national alcohol policy agendas have proliferated across the globe (Babor et al. 2010). Alcohol poses a difficult set of policy challenges for society. Just like tobacco, alcohol is highly addictive (McCambridge and Morris 2019). Alcohol has negative consequences, including for physical and mental health and welfare, for individuals, populations and society. The more alcohol is consumed, the more harm can be expected; this relationship is observed across populations and for a wide range of health- and non-health-related harms (Babor et al. 2010). The policies which are most effective in reducing alcohol-related harms rely on restricting alcohol’s affordability, accessibility, and promotion. In response, some governments have turned to pricing mechanisms, including minimum unit pricing (MUP). MUP imposes a floor price for a standardized dose of alcohol and is designed to discourage the consumption of high-strength, cheap alcohol (Stockwell et al. 2012).

There have been different experiences of alcohol pricing reforms across the UK context. The Scottish government first discussed alcohol pricing in 2008 and enacted legislation for MUP in 2012 (McCambridge, Hawkins, and Holden 2013). MUP has since been debated across UK legislatures. In 2012, the UK government announced its intention to implement MUP but then reversed these plans in the face of industry pressure (Godlee 2013; Gornall 2014). In 2017, the Welsh parliament adopted MUP. MUP legislation took effect in Scotland in May 2018, after delays from legal challenges by the Scotch Whisky Association (SWA), and in Wales in March 2020. In Northern Ireland, support for MUP has been expressed by the government but a lengthy suspension of devolved government, and other policy priorities have slowed progress.

The UK is commonly regarded as a “quasi-federal” political system, with elected assemblies in Scotland, Wales, and Northern Ireland holding constitutionally entrenched powers over key portfolios including health and education (Bogdanor 2003; Gamble 2006). Studies of the policymaking context in the UK post-devolution have tended to focus on Scotland (e.g. Keating et al. 2003; Mooney and Scott 2005; Keating and Stevenson 2006; Cairney 2007; Cairney 2009b; Keating 2010). Less attention has been paid to understanding the policy process in Wales (but see McAllister 2000; Wincott 2005; Royles 2006; Andrews and Martin 2010; Nutley et al. 2012; Royles and McEwen 2015; Chaney, Sophocleous, and Wincott 2020; Lesch and McCambridge 2020) and Northern Ireland (but see Meehan 2012; Birrell 2012).

Research on devolution and its policy implications likely reflects a broader interest in multi-level governance (Bache and Flinders 2004). With authority dispersed to a multiplicity of institutions and actors, scholars have examined processes at the supra-national, regional, and local level (Hooghe and Marks 2003, 2004; Cairney 2012). From this perspective, devolution introduces several competing dynamics. Dispersing legislative authority can enable devolved territories to design policies so that they are better aligned with the preferences and values of local communities (Hooghe and Marks 2004). By the same token, devolution can induce competition between governments, leading to policy innovations, and in some cases policy transfer or convergence (Cairney 2007, 2009b).

Interdependence, policy convergence and policy transfer have been major themes of recent research on multi-level governance and devolution. Researchers distinguish vertical (from national to sub-national level) and horizontal (sub-national to sub-national) variants of policy transfer within federated polities (Evans and Davies 1999; Benson and Jordan 2011). In the UK, Keating and his colleagues (2012) identify three potential pathways: (1) centre to the periphery, (2) periphery to the centre, and (3) across the periphery. The first category is identified as the most common in the UK, largely reflecting Whitehall’s larger size and stronger policy capacity. Horizontal transfer, the third category, has also been observed but is less clearly understood. Notable examples include the elimination of NHS prescription charges and the creation of commissioners for children and the elderly. Whilst these are commonly cited as illustrations of horizontal transfer in the UK (Williams 2005, 2014; Harker 2012), the processes underlying these policy innovations and subsequent transfers have not been systematically examined.

In this article, we seek to contribute to the literature on devolution and horizontal transfer, with a focus on MUP in Wales. This case study is used to better understand how policy ideas may move between jurisdictions, and how this process is shaped by institutional conditions. We argue that whilst policy transfer (from Scotland) is helpful for understanding the origins of MUP in Wales, policy decision-making involved much more than copying or emulation. To explain policy development in Wales, we draw particularly on what Evans (2009) calls hybridization, a type of policy-oriented learning where a policy decision is modelled after a particular example but is also informed by other experiences and insights. We also point towards the role of policy communities as idea carriers, as well as broader developments in the UK and Scotland in shaping the policy decision-making process in Wales.

Our findings hold important implications for research on devolution, policy communities and policymaking in low-capacity settings (Keating, Cairney, and Hepburn 2009; Connell, Martin, and St Denny 2017, 2019). Legal authority, organizational resources and fiscal capacity are commonly identified as key measures of policy capacity (Hood and Margetts 2007; Osterkatz et al. 2016). Low policy capacity within devolved territories is usually regarded as a constraint (Osterkatz et al. 2016; Cole et al. 2021). Our findings additionally suggest that these institutional conditions do not necessarily impede policy innovation and/or policy-oriented learning. Consistent with research in other policy sectors (Connell, Martin, and St Denny 2017), we find that the convening power of the Welsh government enabled policymakers to draw on the knowledge of experts and local stakeholder groups. Our findings suggest that the specific character of Welsh political development, particularly its emphasis on participation and consultation in policymaking (Tewdwr-Jones 2001; Chaney and Fevre 2001), made policy learning possible in this particular lower capacity environment.

The article draws on a range of sources, including primary documents, newspaper articles, and interviews with policy participants, including health officials, civil society actors, elected officials, journalists, and public health experts. Fourteen semi-structured interviews were conducted by the first author in March and April 2019. Interviewees were recruited by e-mail with a response rate of ∼30%, following scoping of the key actors using documentary data sources. Ethics approval for data collection was obtained through the Research Governance Committee at the University York in February 2019. The interviews were transcribed, then thematically coded by ML, with JM supporting the analysis and successive drafts of the findings. The nature of this research design meant that our analysis and conclusions are based on informed interviewees’ perceptions and recollections of policy developments. Accordingly, we have triangulated interview data with government documents and other newspaper coverage in generating key inferences.

In the next section, we bring together literature on policy transfer, political institutions, and devolution in the UK to form the conceptual basis for the empirical analysis. In the third section, we analyse the case study, incorporating a theoretical discussion. The final section reflects on the implications of the findings for future research.

## Theoretical framework

### Policy transfer, political institutions, and policy communities

The concept of policy transfer has a rich history in the study of public policy (for review, see Benson and Jordan 2011). Policy transfer studies explore the “process by which knowledge about policies, administrative arrangements, institutions, and ideas in one political system (past or present) is used in the development of policies, administrative arrangements, institutions and ideas in another political system” (Dolowitz and Marsh 2000, 7).

A continuum of policy transfer processes has been proposed. Building on Dolowitz and Marsh’s (2000) seminal framework, Evans (2009) identifies four types of policy-oriented learning processes: copying, emulation, hybridization, and inspiration. These processes can be distinguished by the extent to which transferred policies deviate from their originals. In instances of *copying*, there is no deviation from the original; policy designs are simply copied and pasted. In *emulation* processes, policies deviate slightly, reflecting minor changes to the policy design. *Hybridization* describes a more labour-intensive process. Here, policymakers fuse a range of insights from different empirical settings to formulate a policy that partly resembles the original. Finally, *inspiration* is where an original policy inspires a government to act but results in a completely distinct policy response. The search for information in the latter two processes is much more resource-intensive. These transfer mechanisms are akin to social learning (Hall 1993) or epistemic learning (Dunlop and Radaelli 2018), where policymakers, experts, and stakeholders collectively puzzle over problems and solutions.

Policymaking institutions may be more or less open to policy transfer. Institutionalist scholarship stresses the impact of formal rules, norms, and historical legacies on political behaviours (Hall and Taylor 1996). Institutions matter for policy, including policy transfer, because these determine where decision-making power is located and circumscribes how it is exercised (Thelen 1999). As Lodge (2003, 163) explains, institutional rules “allocate the roles and responsibilities” and thus “shape [actors’] access and veto power.” Institutional parameters are not necessarily fixed. Changes to political institutions have implications for policy transfer.

Devolution creates new political institutions. One potential consequence of devolution is that it can induce venue-shopping by interest groups (Pralle 2003; Cairney 2007). Politically astute policy communities can adapt to the shifting location of decision-making power by organizing their efforts at multiple levels (i.e. federating their structures) and/or focusing their efforts on one institutional venue, deemed more sympathetic to its interests or ideas. Keating, Cairney, and Hepburn (2009) find evidence of the latter in the UK, some policy communities have shifted their attention to the devolved governments (though this varies across policy sectors). In the Welsh health sector, they describe a “distinct policy community”, comprising health professionals and local organizations keen to advance a “broad public health agenda” (8). Policy communities, or advocacy coalitions (Sabatier 1988), comprise the collection of experts, officials, and advocates that operate in the same policy space. Individuals operating within such communities are bound by a set of shared beliefs, including problem-definitions and policy preferences about solutions (Miller et al. 2011).

Devolution holds potentially profound implications for policy transfer. Creating new sites of policymaking generates opportunities for lesson-drawing (Rose 1991). Policy communities may be well-positioned to facilitate this process, as policy actors are often linked to broader policy communities operating at the regional, national and international level. According to Stone (2004, 550), “the agents of lesson-drawing and policy transfer” tend to be “individuals, networks and organizations.” Policy communities are thus likely important in the policy transfer given their access to and knowledge of a wide range of policy contexts.

### The devolved policymaking context in Wales

Understanding the institutional context in which policymaking unfolds is critical for parsing potential drivers of policy change. Devolution has incrementally led to institutional change in Wales. The first devolved institutions were created with the Government of Wales Act 1998. Since then, the government’s powers have slowly expanded. Initially, the Welsh parliament could only table secondary legislation but following a referendum, it gained primary law-making authority in 2011 (Mitchell 2013; Cole and Strafford 2014). Finally, under the Wales Act 2017, the Welsh parliament moved to a reserved powers model (akin to Scotland), meaning it could legislate on all matters not explicitly reserved to Westminster (e.g. defense, public order, monetary policy).

Both the novelty and incrementalism of devolution have had consequences for policy capacity in Wales. Policy capacity refers to a government’s ability to “review, formulate and implement policies within its jurisdiction” but varies depending on the “quality of resources available” to officials (Fellegi 1996, cited in Wellstead and Stedman 2011, p. 462)*.* In the UK, Whitehall is a highly specialized, well-resourced, and experienced bureaucracy, whilst the devolved administrations are less equipped for policy development and evaluation (Cole, Jones, and Storer 2003; Keating, Cairney, and Hepburn 2009; Cole 2013). Furthermore, there are key differences across the devolved territories. The Scottish bureaucracy is larger than the Welsh one, and the Scottish parliament was initially afforded much greater legislative power (Cole, Jones, and Storer 2003; Cole and Strafford 2014). Royles (2006, 147) describes how the Welsh government experienced “capacity problems” in its early years, citing “inadequate staffing and limited policy development capabilities.” This characterization reflects a broader legacy of asymmetry. Before devolution, the Welsh Office (a branch of the UK Government) largely implemented UK legislation. In contrast, the Scottish Office was more autonomous and thus more experienced in policy development (Jeffery 2006; Royles 2006; McAllister 2000).

From a policy transfer perspective, then, one might anticipate limited policy innovation in Wales, and even a greater likelihood of copying or emulation by the Welsh government. Yet existing research points in the opposite direction. According to one account, Wales has “managed to develop a highly distinct policy agenda in the key areas of health and education” (Bradbury and Mitchell 2005, 301). Innovation has continued over the past decade, most notably with the passage of the Well-being of Future Generations (Wales) Act 2015 (Nesom and MacKillop 2020).

Understanding the inclusion of civil society actors in policy development may help explain how policy innovation is possible in a low-policy capacity environment. Scholars analysing Wales have described it as “more consensual” than Westminster, stressing the “systematic inclusion of pressure participants” in policy development (Cairney 2009a, 361; Entwistle 2006; Chaney and Wincott 2014). The “Welsh Way” describes a consultative approach to policymaking and reflects its small size and the importance of personal relationships between civil society groups and government officials (Keating, Cairney, and Hepburn 2009; Cairney 2008). As Royles and McEwen (2015, 1037) explain:

[S]ub-state governments can overcome limitations of their formal constitutional power … by nurturing and accessing policy networks and expertise to strengthen their policy development.

More recent research has clarified the conditions in which policy communities can promote policy development in low-capacity settings. Connell and his colleagues (2017) use the NATO – Nodality, Authority, Treasure and Organisation – typology of policy tools to emphasize the importance of nodality in Wales. Nodality describes “the property of being in the middle of an information or social network” (44). In the case of homelessness policy, they show how the presence of a dense policy network has been vital for mobilizing policy-relevant knowledge, despite the government’s limited in-house research capacity. This suggests that policy communities may be well-positioned in Wales to frame policy debates and offer expertise to a government whose resources are more limited. Yet, it might also be the case that civil society groups, including experts, are consulted but their capacity for influence might be limited. Determining whether policy communities can influence decision-making is an empirical question and thus must be assessed across each sector and/or policy issue accordingly.

## Case study

### Background

The Public Health (Minimum Price for Alcohol) (Wales) Bill was introduced into the Welsh parliament in October 2017 and passed into law in June 2018. The policy, which took effect in March 2020, imposes restrictions on the sale of alcohol, making it illegal to sell a unit of alcohol below 50 pence.

Alcohol has been a long-standing public health concern in Wales. Wales has seen a steady increase in alcohol-related deaths and hospital admissions in the last 40 years. Alcohol is estimated to cost the government about £76.5 million per annum (Government of Wales 2017). Inexpensive alcohol has been identified as a key driver of consumption and harm. The Welsh experience is consistent with international evidence which has linked harmful levels of drinking to cheap alcohol (Stockwell et al. 2012). As one Welsh politician noted, access to “cheap and strong cider and beer” has made “hazardous and dangerous drinking” much more widespread in Wales.^1^

Interest in alcohol pricing policy prompted several commissioned studies across the UK in the late 2000s. In 2008, the UK Department of Health had experts at the University of Sheffield model different price-based policy interventions (Hawkins and McCambridge 2019). Subsequent work on the Sheffield Alcohol Policy Model demonstrated the potential impact of introducing MUP in Scotland (Purshouse et al. 2009) and England (Purshouse et al. 2010).

### Devolution, alcohol policy and the UK context

Alcohol policy in Wales is shaped by devolution. Taxation is not a devolved power and so the ability of the Welsh (and Scottish) governments to influence alcohol prices is circumscribed by the constitution. Since taxes could not be raised by the government, “[Wales had] to look to other levers, including minimum unit pricing”.^2^ In 2009, with alcohol-related deaths on the rise, the Welsh government pressed Westminster to increase taxes on alcohol (Hutchinson 2009). In 2012, the UK government announced it would adopt MUP for England and Wales but then reversed course a year later.

Alcohol policy in Wales is further shaped by the UK’s asymmetrical approach to devolution, reflecting the 1997 referenda results in Scotland and Wales on whether devolution should occur at all. Unlike Scotland where approximately three-quarters of voters supported devolution, in Wales, the result was a very narrow majority, and the parliament did not function as a primary law-making body until 2011 (Cole and Strafford 2014).

Uncertainty over whether the parliament possessed the legal authority to adopt minimum pricing for alcohol complicated the policy process. Under devolution, the Welsh parliament’s powers to legislate on alcohol were “somewhat ambiguous”.^3^ Between 2014 and 2015, the Welsh government contemplated adopting its own MUP but faced pushback from UK-level officials. In 2016, several MPs questioned the Welsh parliament’s authority to legislate an MUP. The Welsh Secretary (UK Government cabinet member responsible for Wales) told a reporter that because “alcohol is so associated with criminal justice”, alcohol pricing should be considered as a UK-level responsibility (BBC News 2016). In contrast, Welsh government officials maintained they possessed legislative competence, specifically under the Government of Wales Act 2006, which granted authority over the “[p]romotion of public health” (Government of Wales 2017).

Further complicating the matter were evolving changes to the Welsh government’s powers. As noted above, in 2017, Wales moved to a reserved powers model, meaning specific powers would be reserved for Westminster (most importantly here including alcohol pricing). For proponents of MUP, these macro constitutional changes posed a major threat to the prospect of policy change in Wales.^4^ Yet some public health experts and government lawyers also saw the impending change as a potential opportunity for action. The provisions of the Wales Act did not take effect immediately and so this provided a fleeting “window of opportunity” to introduce enabling legislation (Livingston et al. 2020, 2). Despite the potential risk of a legal challenge by the UK government, the Welsh government followed this advice and introduced the legislation.

A second complicating factor was the legal challenge to the Scottish law by alcohol producers. The SWA claimed the measure infringed both UK legislation and the EU’s single market law (Hawkins and McCambridge 2020b). Had the courts accepted the SWA’s argument, MUP would be unlawful in Wales as well. Ultimately, both European and UK courts rejected the alcohol industry’s argument, deeming MUP as a justifiable use of public health legislation. The final ruling, however, was not made until November 2017, leaving Welsh actors to operate in a context of considerable uncertainty. The court challenge received considerable attention in the Welsh press (Rutherford 2015; Smith 2017, 2018) and was identified by every interviewee as a salient issue (see below). Critically the Welsh parliament passed MUP legislation some weeks ahead of the final court ruling in Scotland (see Table 1). This indicates that whilst Welsh policymakers were mindful of the uncertainties about the direction of the court’s ruling, they were not dissuaded by it from progressing the legislation.

**Table 1.** UK MUP-related timelines of events.

Date	Event
June 2008	Scottish government announces plan to adopt of MUP
March 2012	UK government announces MUP will be part of a new alcohol strategy
June 2012	The Alcohol (Minimum Pricing) (Scotland) Act 2012 is adopted by Scottish parliament
July 2012	Scottish Whisky Association (SWA) challenges legal basis of Scottish MUP
July 2013	UK government backtracks on plan to adopt MUP
April 2014	Welsh government announces interest in MUP as part of its Public Health (Wales) Bill
July 2015	Welsh government separates MUP from the Public Health (Wales) Bill and issues draft Public Health (Minimum Price for Alcohol) (Wales) Bill
September 2016	The Welsh Secretary (UK) argues alcohol pricing is not a devolved matter
October 2017	The Public Health (Minimum Price for Alcohol) (Wales) Bill is introduced into the Welsh parliament
November 2017	The Supreme Court of the United Kingdom rejects SWA’s legal challenge, upholding MUP legislation in the UK
August 2018	Welsh parliament enacts Public Health (Minimum Price for Alcohol) (Wales) Act 2018.
May 2018	Scottish MUP is implemented
March 2020	Wales MUP is implemented

### Scotland, MUP, and agenda-setting

Interviewees stressed the range of evidentiary sources that were part of the decision-making process to ultimately adopt MUP in Wales. As one advocate explained, the Scottish legislative debate over MUP played an agenda-setting function, as the initial debates over the bill in the early 2010s were “closely followed by public health advocates in Wales.”^5^ One public health expert recalled having discussions with civil servants about MUP while it was being debated in Scotland:

I remember when I was putting together our public health report in 2012, I had discussions with some of the civil servants in Welsh government and they said, ‘we know, [MUP] is on our radar.’ [Wales] is pretty good at looking either across the UK or internationally about what’s going on and [they] were very aware of what was happening in Scotland.^6^

Another interviewee explained how this reflected a broader pattern in Welsh politics:

Wales is a small country, it has limited [policy] infrastructure … Scotland is much bigger so often [the Scottish government] will [take] the lead on a number of policy areas and Wales will [follow] quite closely behind.^7^

Among some interviewees, there was reluctance, however, to describe the Scottish legislation as the inspiration for Wales. As one government official explained:

Obviously, the fact that the legislation being taken forward in Scotland was a factor … but I think probably both countries were exploring similar issues, just in terms of levels of consumption, things like alcohol-related deaths [and] hospital admissions.^8^

According to one politician, MUP discussions between the two governments were actually quite minimal. As they explained:

[The Welsh and Scottish] governments are [just] beginning to talk to one another [about MUP now] … When we implement it [we’ll talk through] all the hiccups and stuff but this was less so around the specific decision [to adopt MUP].^9^

The most salient piece of information for Welsh policymakers seemed to be the legal challenge to the Scottish legislation. As one politician recalled:

I certainly remember discussions that we had during the deliberations of the bill … wanting to wait and see how it works in Scotland before [MUP was] actually implemented in Wales. But the main point of interest was also whether the Scottish government was [legally] allowed to do it.^10^

These characterizations are consistent with evidence from primary documents. In the Welsh government’s explanatory memorandum on MUP, Scotland, Canada, Ireland and Northern Ireland’s experiences with alcohol pricing legislation are all described but no special prominence is given to Scotland (Government of Wales 2017). One factor that likely constrained capacity for learning from Scotland was the legal challenge, which prevented implementation, and by extension, evaluation.

The design of MUP in Wales is remarkably similar to Scotland’s policy. In both jurisdictions, the minimum price is set at 50 pence per unit of alcohol. Furthermore, both are subject to 5-year sunset clauses, requiring governments to reauthorize the legislation following evaluation of impacts. There are some marginal differences in the operation of the two policies. The Scottish legislation includes broader restrictions on alcohol retail promotions (Livingston et al. 2020) and the enforcement protocols vary in each country (Government of Scotland 2018, 2020). The overall similarities in approaches to alcohol pricing, however, would seem to suggest that Wales copied Scotland. Yet as the subsequent discussion illustrates, the transfer process was far more complicated, revealing how a range of actors and the institutional context promoted hybridization.

### The public health policy community and hybridization

Interview data illustrate the central role of the public health community in fostering policy change. The Welsh government first gave serious consideration to MUP in 2014. During early discussions about a wide-ranging Public Health Bill, public health advocates identified access to cheap alcohol as a pressing issue and cited the Scottish policy as a potential model.^11^

Between 2014 and 2017, the Welsh government examined the merits of a new policy on alcohol pricing. In 2014, the government had tasked an external Advisory Panel on Substance Misuse (APoSM) with reviewing the international evidence on MUP as well as other policy measures. The bulk of the evidence came from modelling data and international research on pricing. The panel examined the Scottish decision as well as price floors for alcohol in Saskatchewan and British Columbia.^12^ Panelists used their contacts from the Scottish public health community to help interpret the evidence.^13^ Using data from Public Health Wales, the panel also studied the magnitude of alcohol-related harms in Wales. In its final report, the panel urged the government to follow Scotland’s lead on MUP (Advisory Panel on Substance Misuse 2014). According to one health official, the legal uncertainty, coupled with potential objections from Westminster, led the government to remove MUP from its initial 2015 Public Health bill and make the legislation a standalone bill.^14^

Public health had been a devolved power in Wales since the creation of the Welsh parliament. Welsh advocates with a keen interest in advancing population-level policies thus focused their efforts at this level of governance. The emphasis placed in the Welsh parliament on stakeholder engagement (Keating, Cairney, and Hepburn 2009; Chaney and Fevre 2001) has enabled these actors to form relationships with civil servants and politicians. As one advocate explained:

We’re close to the Welsh government and that can be a help or a hindrance … But when you’re all on the same page and you’ve all got a will to make things happen you can get some of the change that you need. That’s one of the aspects which has led to the positive work on minimum unit price in Wales.^15^

The early framing of alcohol as a public health issue had important consequences, placing the public health community as a key authority on the bill. Alcohol pricing policy was relatively new terrain for the Welsh government, leaving advocates in a “strong position to influence civil servants and ministers.”^16^ As one advocate recalled:

The Welsh government [didn’t] have a huge amount of capacity to understand alcohol policy in-house. A lot of that [work] was farmed out to Alcohol Concern Wales, [a leading alcohol advocacy group in Wales] and other expert groups.^17^

In April 2014, whilst APoSM’s evidence review was being undertaken a Public Health White Paper was released. The White Paper described MUP as a “public health” measure that could help reduce alcohol-related harm (Government of Wales 2014, 31). Framing MUP as a public health measure may have been strategic on the government’s part. Yet, as one advocate explained, the government’s framing likely reflected a genuine concern about alcohol and public health:

I think the Welsh government and their medical advisers have been very keen to see [alcohol] as a public health issue, and I think they do the same with illegal drugs as well.^18^

The public health community was not restricted to public health experts in Wales. In 2014, the Welsh government commissioned the Sheffield researchers to model the impact of different minimum prices (Government of Wales 2014). As another health official recalled:

We’d seen the modelling work that they had done both for England and Scotland … so we had an iteration of the model in 2014 and then we updated that in 2017 … Because [MUP] hadn’t been implemented anywhere there [wasn’t] evaluation evidence that we [could] rely on. The best we have at this point is the modelled evidence.^19^

According to the modelling, illness, crime and workplace absenteeism could be significantly reduced with a 50-pence minimum price (Meng et al. 2014). The Sheffield Model thus enhanced the attractiveness of this particular policy measure for a government with limited policy capacity. As another health official explained:

I think the fact that we did have a model that had used data specifically for Wales was a strength … Sometimes we don’t always have the data and the evidence at the Wales level, so you’re trying to generalise from evidence in other jurisdictions, other countries, and possibly other cultures sometimes as well. Whereas at least with modelling … as much of the data as possible was Wales-based data.^20^

As one advocate explained, the Sheffield research was attractive because it presented decision-makers with a clear policy solution:

From a policy study perspective, it was like, this is the problem and [MUP] is the solution. This is how you introduce the solution and this is what the solution will produce. It was all there on five slides that could you get from [the Sheffield Alcohol Group website] or via any number of conference report presentations.^21^

There were also close ties between the Welsh public health community, UK public health groups, and experts, including the Sheffield researchers. Interviewees identified key umbrella organizations, including the UK-based Alcohol Health Alliance which could “point interested parties to ready-made solutions around pricing, availability and marketing.”^22^ One advocate stressed the density of the alcohol policy network:

[Welsh health officials] had no lack of opportunity to go to conferences and meetings where people would present on [MUP] … It would have been almost impossible for someone working with that alcohol brief to not have MUP somewhere near the top of their inbox. MUP was almost the only thing people were talking about for two years.^23^

Consultations provided another key opportunity for public health advocates to frame key issues for policymakers. The Welsh government undertook several consultations with stakeholders: first in 2014 following its proposal to adopt MUP, second, between July and December 2015 to discuss design, and finally in 2018 after the passage of the legislation to consult on pricing level. At each point, public health advocates and organizations, including Alcohol Concern Cymru/Wales, Alcohol Health Alliance UK, the Association of Directors of Public Health, and several prominent medic organizations, used these opportunities to marshal evidence in favour of MUP.

In October 2017, the government referred the bill to the Health, Social Care, and Sport Committee to provide greater scrutiny and further consultation. Written responses and oral evidence came disproportionately from public health advocates and their allies, including the Sheffield researchers, Public Health Wales, and the Welsh NHS Confederation. Testimony included references to Scotland’s decision, discussions of the Sheffield model, and health-related outcomes in Canadian provinces with a price floor. In its final report, the committee supported the government’s approach, citing the testimony of public health experts, as well as offered some amendments for strengthening parts of the legislation (National Assembly for Wales 2018). Thus the committee proceedings provided another key opportunity for the public health policy community to brief legislators on the evidence base for MUP.

### The role of opponents in the policy process

The deep involvement of public health advocates in the policy process contrasts significantly with that of the alcohol industry. Although policymakers anticipated resistance, interview data and media coverage between 2014 and 2017 suggest industry opposition was relatively muted. Numerous UK-level alcohol industry groups voiced objections to the bill during the committee’s scrutiny work (National Assembly for Wales 2018). Compared to Scotland and England, however, the alcohol industry was much less prominent in the process. As one politician remarked: “For whatever reason, [the alcohol industry] didn’t engage so much with us.”^24^ This appears consistent with research on organized interests in Wales, which suggests that some business groups have been “slow to strengthen” their political presence in Cardiff (Keating, Cairney, and Hepburn 2009, 7). Alternatively, it might reflect the fact that, unlike Scotland and England, Wales is not a major alcohol producer. As one advocate explained:

The alcohol industry is far [better] organized, and actually just far better plugged into the lobbying networks [in Westminster] … [and so] there's a degree to which [industry] just weren’t looking at Cardiff.^25^

Another potential explanation is that the industry actors were strategically focusing their efforts on legal challenges mounted elsewhere; first in Scotland and later at European Union (EU) and UK levels (Hawkins and McCambridge 2020a). Although alcohol industry actors did participate in the consultations led by the Welsh government, they were simply less vocal and prominent in Wales compared to the Scottish and English cases (Holden and Hawkins 2013; McCambridge, Hawkins, and Holden 2014).

It’s also worth noting which actors did not ultimately oppose MUP in Wales. Despite early indications, the UK government never issued any legal challenge to Wales’s legislative competence. This might be partly explained by the UK government’s focus on other issues such as Brexit, and by the complexity of the legal and political issues which saw the UK Government as representing Scotland’s right to pass MUP as public health legislation in the EU court case, whilst not proceeding with this particular policy for England (Hawkins and McCambridge 2020a). According to one advocate:

[I think] one of the reasons the UK government didn’t get involved in the final stages was because they [were] far too busy … getting us completely in or out of the European Union.^26^

## Discussion

Our analysis shows that alcohol policy innovation in Scotland was a necessary but insufficient condition for alcohol policy change in Wales. Both countries adopted very similar policies yet it would be a mistake to conceptualize the Welsh decision as a case of copying or emulation. Our analysis documents a process of policy-oriented learning, where policymakers made a concerted effort over time to draw on an assortment of expertise and experiences after the Scottish government’s policy sparked interest in Wales. For those reasons, MUP in Wales is more consistent with what Evans terms hybridization; the policy is not a carbon copy but shares many similarities to the original. Public health actors operated as the key agents of hybridization in the policy process, drawing on a wealth of policy-relevant knowledge, transnational networks and familiarity with the Welsh political system.

Our analysis also suggests that broader developments in the UK, including ambiguity over legislative competence as well as the Scottish legal challenge, influenced the policy process in Wales, including the time taken. Although the Scottish model was a key consideration for Welsh decision-makers, wider political developments in Scotland and the UK also influenced the process. Since the principal legal question in the challenge to the Scottish MUP law centred on whether the policy constituted a justifiable public health protection measure, this made it much easier for the Welsh government to maintain its preferred framing of the policy (i.e. as a public health measure), which was the same as in Scotland (Katikireddi, Bond, and Hilton 2014). Making MUP a stand-alone bill was in part motivated by the impending legal case in Scotland. The protracted nature of that dispute afforded greater time for the public health community to assemble a stronger evidence base in favour of the legislation and thus promote learning. Indeed the legal challenges by industry and consequent delay in implementation in Scotland appear to have encouraged parallel processes in which public health arguments and evidence were seen to be needed to be strengthened by the policy community, both in Scotland (Hawkins and McCambridge 2020a) and in Wales. These processes were also connected to some degree, as some of the same actors e.g. the Sheffield researchers, were involved in both. Questions of legislative competence and the potential challenge of the UK government lingered as a major concern for the Welsh government. Together, these consequences suggest that policymaking at the devolved level should be studied with a multi-level governance lens. That being said, alcohol pricing might be somewhat atypical in the influence of other governments and exogenous institutional processes on devolved policymaking. On the other hand, the involvement of well-resourced transnational corporations with capacity for venue shopping, and the very complexity of the policy processes involved, may make alcohol policy analysis resonate with political scientists focused on better understanding the interplay between levels of governance in strongly contested and challenging areas of policy development.

We also show how the specific design of governance structures in Wales facilitated hybridization. Welsh policymakers had access to key experts and stakeholder groups through various fora, including expert committees, consultations and committee proceedings. Our analysis shows that over the course of devolution, public health actors have strategically embedded themselves into policymaking structures in Wales. By contrast, industry actors have focused on maintaining their relationship-building efforts and political activities at the UK-level. Relationships with legislators, familiarity with the Welsh political system, and the government’s promotion of inclusivity and stakeholder engagement meant that public health advocates were well-positioned to promote their preferred problem-definition and policy alternatives (e.g. the Scottish model). Incentives for policy transfer operate within institutional contexts with clear sets of norms and ideas about how policy should be developed, and on what. Rather than lower policy capacity inhibiting innovation, the experience of the UK devolved administrations on MUP and alcohol has been that policymakers have been willing to take on challenging policy issues that damage population health. A cursory comparison of the Scottish and Welsh policies is unlikely to pick up on these nuances of the policy process in Wales. Thus this study demonstrates the value of analysing a single-case study over an extended period of time.

The influence of the public health policy community must also be appreciated within the context of the Welsh government’s limited policy capacity. We find that policymakers turned to the public health community precisely because they lacked experience in formulating alcohol pricing policy. Different parts of the policy community helped close key information gaps for decision-makers, drawing attention to the local harms of alcohol use (Alcohol Concern Cymru/Wales), the international evidence on pricing (Alcohol Health Alliance, APoSM), and the estimated effects of different pricing scenarios on health and social outcomes (Sheffield researchers). Our analysis attests to the importance of policy communities, as has also been recently demonstrated in the passage of alcohol public health legislation in Ireland (Lesch and McCambridge 2021a, 2021b). This finding contributes to a growing body of work suggesting that the policy tools of government, and particularly subnational government, are not necessarily restricted to fiscal capacity and/or legal authority in interesting ways (Connell, Martin, and St Denny 2017, 2019). It is to be expected, however, that willingness to draw on external participants’ ideas may vary across sectors and/or issues. If governments possess more experience and organizational resources internally in a particular policy sphere they might be less inclined to turn to civil society groups and experts.

## Conclusion

Our findings shed light on interesting issues for the future study of policy transfer, multi-level governance and alcohol policy. Although we know that policy-oriented learning operates along a spectrum (from copying to inspiration), we have a less clear understanding of the conditions that lead to one mechanism over another. Our analysis suggests that learning is a function of policy capacity (broadly defined) as well as specific norms that guide policy development and participation in the process. Future work will be required to better understand the linkages between learning and the configuration of institutions.

The study’s findings should also be considered within the broader literature on multi-level governance. Public support for devolution has been historically less pronounced in Wales than in Scotland (Jones and Scully 2003). More recent studies, however, suggest that the Welsh’s government legitimacy is increasing (Scully and Wyn Jones 2015). Heightening the visibility of government actions through policy can strengthen the bonds between citizens and the state (Mettler and Soss 2004). Adopting Welsh approaches to public health could produce self-reinforcing feedback effects of this nature; MUP has been a means by which the “Welsh Way” can be communicated. Research that incorporates attention to sub-state nationalism (Béland and Lecours 2008) might be particularly instructive for exploring future dynamics.

Finally, the findings also have implications for the study of alcohol policy. Many evidence-based measures impose significant constraints on business practices and are challenging to have adopted (McCambridge, Mialon, and Hawkins 2018). Particularly in producer countries, including the UK, industry actors are well organized politically. To date, alcohol policy researchers have focused on the industry’s success in resisting evidence-based policies through framing ideas and involvements in policy processes (Babor and Robaina 2013; McCambridge, Mialon, and Hawkins 2018). This analysis demonstrates the importance of studying broader institutional contexts and focusing on the resulting policy outcomes (Lesch and McCambridge 2020), and identifying where industry actors are not well organized or embedded in policy networks.

## Notes

1 Interview A. Welsh Assembly Member, Cardiff, Wales, 27-Mar 2019.

2 Interview B. Civil servant, telephone interview, 12-Apr 2019.

3 Interview C. Journalist, Cardiff, Wales, 26-Mar 2019.

4 Interview D. Member of Parliament, Cardiff, Wales, 23-Apr 2019.

5 Interview E. Advocate, Cardiff, Wales, 25-Mar 2019.

6 Interview F. Public health expert, telephone interview, 12-Apr 2019.

7 Interview G. Public health expert, Cardiff, Wales, 15-Apr 2019.

8 Interview B.

9 Interview A.

10 Interview H. Welsh Assembly Member, Cardiff, Wales, 26-Mar 2019.

11 Interview E.

12 Interview I. Public health expert, Cardiff, Wales, 25-Mar 2019.

13 Ibid.

14 [14] Interview B.

15 Interview E.

16 Interview J. Advocate, telephone interview, 22-Mar 2019.

17 Ibid.

18 Interview E.

19 Interview H.

20 Interview B.

21 Interview J.

22 Interview E.

23 Interview J.

24 Interview H.

25 Interview K. Advocate, Cardiff, Wales, 15-Apr 2019.

26 Interview E.
